# Characteristics and risk factors for typhoid fever after the tsunami, earthquake and under normal conditions in Indonesia

**DOI:** 10.1186/1756-0500-3-106

**Published:** 2010-04-17

**Authors:** Agung Budi Sutiono, Andri Qiantori, Hirohiko Suwa, Toshizumi Ohta

**Affiliations:** 1 The University of Electro-Communications, Graduate School Information Systems, Graduate Department Social Intelligence and Informatics, 1-5-1 Chofugaoka, Chofu-shi, Tokyo, 182-8585 Japan; 2 Hasan Sadikin Hospital, Padjadjaran University Faculty of Medicine, Jl. Pasteur 38 Bandung, 40161 Indonesia

## Abstract

**Background:**

Although typhoid transmitted by food and water is a common problem in daily life, its characteristics and risk factors may differ in disaster-affected areas, which reinforces the need for rapid public health intervention. Surveys were carried out post-tsunami in Banda Aceh, post-earthquake in Yogyakarta, and under normal conditions in Bandung, Indonesia. Logistic regression analysis was used to assess the risk factors with the dependent variable of typhoid fever, with or without complications.

**Findings:**

Characteristic typhoid fever with complications was found in 5 patients (11.9%) affected by the tsunami in Aceh, 8 (20.5%) after the earthquake in Yogyakarta, and 13 (18.6%) in Bandung. After the tsunami in Aceh, clean water (OR = 0.05; 95%CI: 0.01-0.47) and drug availability (OR = 0.23; 95%CI: 0.02-2.43) are significant independent risk factors, while for the earthquake in Yogyakarta, contact with other typhoid patients (OR = 20.30; 95%CI: 1.93-213.02) and education (OR = 0.08; 95%CI: 0.01-0.98) were significant risk factors. Under normal conditions in Bandung, hand washing (OR = 0.07; 95%CI: 0.01-0.50) and education (OR = 0.08; 95%CI: 0.01-0.64) emerged as significant risk factors.

**Conclusion:**

The change in risk factors for typhoid complication after the tsunami in Aceh and the earthquake in Yogyakarta emphasizes the need for rapid public health intervention in natural disasters in Indonesia.

## Background

On December 26, 2004, an earthquake that measured 9.0 on the Richter scale occurred 150 km off the coast of Sumatra-Indonesia in the Indian Ocean, and triggered a widespread tsunami that hit Aceh 45 minutes later and devastated an 800-km coastal strip. Approximately 130,000 people died, and a further 37,000 went missing presumed dead [1]. On May 27, 2006, an 5.9 on the Richter scale earthquake struck about 25 km south-southwest of Yogyakarta city, which affected 36,299 people, with 5782 fatalities, and caused damage to 135,000 homes [2,3].

Infectious diseases are an additional problem in disaster-affected areas [4]. Typhoid fever may cause serious complications after a disaster. It is a water-borne disease due to contaminated S. typhi in human excreta and transmitted via hands [5]. Post-disaster typhoid fever outbreaks were reported in Puerto Rico following Hurricane Betsy in 1956 and in Mauritius following a cyclone in 1980 [6]. The biggest epidemic of typhoid fever following a complex disaster in two decades occurred in Tajikistan in 1992-1997, during which, around 21,000 internally displaced persons (IDPs) were created [7]. After a natural disaster in Calamba, near Manila, Philippines in March 2008, approximately 1400 people displayed typhoid symptoms, with the most serious complications being intestinal bleeding and typhoid perforation [8].

During these disasters, there are usually disruption of availability of clean water for food preparation and hand washing as well disruption of existing medical services [4,9]. The characteristics and risk factors of typhoid fever with complications in tsunami and earthquake might have different risk factors compared with the normal situation. The analysis, surveillance and control of risk factors for infectious diseases are important functions in public health [10,11]. The lack of facilities, difficult access to health services, and changing risk factors emphasize the need for rapid public health intervention to prevent typhoid fever in disaster-affected areas.

## Methods

### Study area

**Figure 1:**
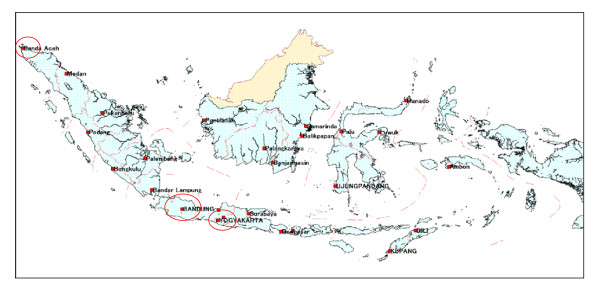
**Study area typhoid fever with complications in post-tsunami (Aceh), post-earthquake (Yogyakarta) and normality (Bandung), Indonesia**. Source: Geology and oceanography research centre, Indonesia.

Typhoid fever patients were interviewed in three districts: post-tsunami Banda Aceh; post-earthquake Bantul, Yogyakarta; and Bandung under normal circumstances (Figure 1). Demographically, Banda Aceh city is occupied by 220,737 inhabitants with a population density 4.25 per km^2 ^[12,13]. Bantul county, Yogyakarta has 813,087 inhabitants in an area of 3186 km^2^, with a population density of 255.21 per km^2 ^[12]. Bandung city has 2,510,982 inhabitants in an area of 34,597 km^2^, with a population density of 72.58 per km^2 ^[14].

### Variables definition

We defined the independent variables based on a previous study, namely age, sex, educational level, clean water, hand washing, contact with other typhoid patients [15], and typhoid complications/no complications as dependent variables. Educational level was classified as primary school (up to junior high school level, which is compulsory in Indonesia [16]), and secondary school (senior high school level and above). Rechecking variables is depending on patient behavior. Doctors suggest that patients submit themselves for rechecking after being discharged home, and it is up to the patients to decide to go. Drug availability seems to be a problem in disaster areas, in relation to where patients can obtain the required drugs and in administering them correctly. The first drugs of choice for *S. typhi *are chloramphenicol, ampicillin, ciprofloxacin and third-generation cephalosporins [17]. Since typhoid fever is water-borne, clean water is important, and drinking water from sources such as wells and municipal water points should be boiled before being drunk. Clean water checking involves ascertaining whether there is a chlorination program, and bacterial examination of water samples in the disaster-affected area. Patients should wash their hands in clean water before eating [18]. Contact with other typhoid patients refers to contact time of > 14 days when patients stayed with relatives/friends or with a typhoid patient and shared food from the same plate [19]. Typhoid complications refer to the manifestations of typhoid fever, whether intra- and/or extra-intestinal (such as intestinal bleeding, typhoid perforation, toxic typhoid, and typhoid encephalopathy) [20-23].

### Data collection and selection criteria

**Figure 2:**
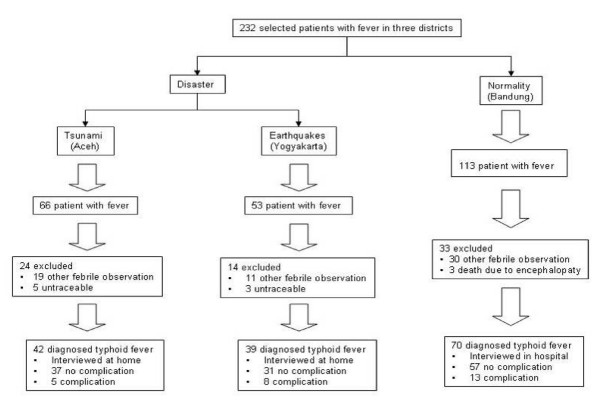
**Selection criteria typhoid fever with complications**.

Permission to perform the study was obtained from Zainoel Abidin Hospital in Banda Aceh, Bantul Health Office in Yogyakarta, and Hasan Sadikin Hospital, Bandung. Typhoid fever was confirmed by positive blood/bone marrow/feces culture for *S. typhi *[19]. Patient data were taken from their medical records, and a survey was subsequently conducted to verify the disaster situation. The questionnaire was developed based on previous studies [15,19,24] and tested in small samples (20 individuals from Banda Aceh, Yogyakarta and Bandung). The selection criteria flowchart is shown in Figure 2. Informed consent was obtained from the respondents. All respondents answered our questionnaire after giving their informed consent. For questions to children aged < 13 years, responses were given by their mother or other family members, who were fully aware of the condition of their children during hospitalization and discharge. Brief training and explanation of the study were given to the students from Syiah Kuala University Banda Aceh, Gajah Mada University Yogyakarta, and Padjadjaran University Hasan Sadikin Hospital, Bandung before surveillance and interviewing respondents. Socio-cultural issues and customs vary between provinces in Indonesia, and this had to be taken into account. Data were stored anonymously and confidentially.

### Interview

This interview was conducted because of the lack of disaster information in medical records. The participants in all the three groups responded to the same questionnaire after giving their informed consent. The responses of the patients were used to obtain a full description of the disease and disaster situation. The questionnaires were used to guide the interviewers to explore the patients' experiences retrospectively. Sample questions were as follows:

• "Did the doctor suggest that you to go for a check-up after recovery?"

• "Did you go for a check-up as the doctor suggested?"

• "Did you obtain the required drugs and take them correctly?"

• "Did you obtain clean water and check that it was clean?"

• "Did you wash your hands before eating?"

• "Did you have contact with other typhoid patients?"

The responses were coded as 0 for "no" and 1 for "yes".

### Statistical analysis

The typhoid variables from the questionnaires were entered on a Microsoft Excel 2003 spreadsheet, and SPSS version 17.0 was applied for logistic regression analysis to determine the significant independent risk factors for typhoid complications in each district.

## Results

In Aceh, data were collected 2 months after the tsunami, in Yogyakarta, 1 month after the earthquake, and in Bandung, data were collected from August 2006 to January 2007. There was different time duration due to it was related to the rehabilitation and reconstruction phase after disaster compare to the normal situation. We found 232 patients with fever in the three districts. There were 66 post-tsunami patients in Banda Aceh. Nineteen patients were excluded because of negative blood/bone marrow/feces culture for *S. typhi*, and 5 were untraceable because of an unclear address, or they had moved house. Therefore, the final number of patients interviewed was 42, including 5/42 (11.9%) with typhoid complications. A similar pattern emerged in Yogyakarta, where there were 53 patients with fever; 14 were excluded and 39 were interviewed, including 8/39 (20.5%) with typhoid complications. Under normal conditions at Bandung, we found 113 cases of typhoid, with 33 patients excluded and 70 interviewed, including 13/70 (18.6%) with complications (Figure 2). Three patients died from typhoid encephalopathy due to came to the hospital in the late stage of illness.

The subject characteristics for typhoid cases in the three districts are presented in Table 1. The mean ages in Aceh, Yogyakarta and Bandung were 36.1 ± 15.4 years (interquartile range 26), 35.5 ± 14.5 years (interquartile range 21) and 19.3 ± 13.7 years (interquartile range 54), respectively. In the Aceh tsunami and the Yogyakarta earthquake, the number of male patients dominated, with 25 (59.5%) and 20 (56.4%), respectively. On the other hand, Bandung had more female patients, at 42 (60.0%). Complications emerged in 5 cases, 4 male (9.5%) and 1 female (2.2%) after the Aceh tsunami, 8 cases with 5 male (12.8%) and 3 female (7.7%) after the Yogyakarta earthquake, and 13 cases with 4 male (5.7%) and 9 female (12.9%) in Bandung under normal circumstances.

**Table 1:** Characteristic of typhoid fever patients in three districts

Variables	Classification	Tsunami (Aceh) n = 42 [%]	Earthquake (Yogyakarta) n = 39 [%]	Normality (Bandung) n = 70 [%]
Age [years]	Mean ± SD Interquartile range	36.1 ± 15.4 26	35.5 ± 14.5 21	19.3 ± 13.7 54
Gender	Male Female	25 [59.5] 17 [40.5]	20 [56.4] 19 [43.6]	28 [40.0] 42 [60.0]
Education	Primary Secondary	13 [31.0] 29 [69.0]	22 [56.4] 17 [43.6]	47 [67.1] 23 [32.9]
Complications	Male Female	4 [9.5] 1 [2.2]	5 [12.8] 3 [7.7]	4 [5.7] 9 [12.9]
Recheck		38 [90.5]	36 [92.3]	64 [91.4]
Go to recheck		30 [71.4]	30 [76.9]	51 [72.9]
Drug availability		28 [66.7]	28 [71.8]	50 [71.4]
Clean water		34 [81.0]	32 [82.1]	58 [82.9]
Water checking		10 [23.8]	9 [23.1]	15 [21.4]
Hand washing		38 [90.5]	35 [89.7]	64 [91.4]
Contact with other typhoid		19 [45.2]	17 [43.6]	36 [51.4]

Table 2 describes the type of complications that occurred post-tsunami in Aceh, post-earthquake in Yogyakarta, and under normal circumstances in Bandung. In Aceh, the complications included 2 cases of intestinal bleeding, 2 of typhoid perforation and 1 of toxic typhoid. In Yogyakarta, we found 4 cases of intestinal bleeding, 1 of typhoid perforation, and 3 of typhoid encephalopathy. In Bandung, there were 2 cases of intestinal bleeding, 3 of typhoid perforation, 4 of typhoid encephalopathy, and 4 of toxic typhoid. Patients with intestinal bleeding explained that their chief complaints included occult bleeding in their stools and acute abdominal symptoms associated with typhoid perforation. Meanwhile, the chief complaint of patients with typhoid encephalopathy and toxicity was a loss of consciousness.

**Table 2:** Type of complications on typhoid fever

Post tsunami (Aceh) n = 5	Post earthquake (Yogyakarta) n = 8	Normality (Bandung) n = 13
2 Intestinal bleeding	4 Intestinal bleeding	2 Intestinal bleeding
2 Typhoid perforation	1 Typhoid perforation	3 Typhoid perforation
1 Typhoid Toxic	3 Typhoid Encephalopaty	4 Typhoid Encephalopaty
		4 Typhoid Toxic

In Table 3, bivariate correlation analysis showed that education (p = 0.01), drug availability (p < 0.01) and clean water (p < 0.01) were significant for typhoid complications after the tsunami in Aceh. After the earthquake in Yogyakarta, education (p = 0.05) and contact with other typhoid fever patients (p < 0.01) were found to be significant. Under normal conditions in Bandung, the significant bivariate correlation was for education (p = 0.05), drug availability (p = 0.03) and hand washing (p = 0.01).

**Table 3:** Bivariate correlation analysis with grouping variable complication and non complication

	Tsunami (Aceh)				Earthquake (Yogyakarta)				Normality (Bandung)			
	
Variables		Mean	95% CI			Mean	95% CI			Mean	95% CI	
									
	Sig.	diff	Lower	Upper	Sig.	Diff	Lower	Upper	Sig.	diff	Lower	Upper
Age	0.33	0.23	-0.25	0.71	0.49	0.14	-0.27	0.55	0.46	-0.11	0.15	-0.42
Gender	0.84	-1.48	-16.53	13.57	0.22	-6.97	-18.30	4.36	0.87	0.67	4.25	-7.81
Education	**0.01**	**0.56**	**0.14**	**0.98**	**0.05**	**0.39**	**0.00**	**0.78**	**0.00**	**0.40**	**0.14**	**0.13**
Recheck	0.41	0.12	-0.17	0.41	0.58	0.06	-0.16	0.28	0.34	0.08	0.09	-0.09
Go to recheck	0.66	-0.09	-0.54	0.35	0.29	0.18	-0.16	0.52	0.32	0.14	0.14	-0.14
Drug availability	**0.00**	**0.76**	**0.36**	**1.15**	0.28	-0.19	-0.56	0.17	**0.03**	**0.31**	**0.14**	**0.04**
Clean water	**0.00**	**0.92**	**0.67**	**1.17**	0.57	0.09	-0.23	0.40	0.54	0.07	0.12	-0.16
Water checking	0.19	0.27	-0.14	0.68	0.44	0.13	-0.21	0.48	0.56	0.07	0.13	-0.18
Hand washing	0.41	0.12	-0.17	0.41	0.82	0.03	-0.22	0.28	**0.00**	**0.27**	**0.08**	**0.11**
Contact with other typhoid	0.81	0.06	-0.43	0.55	**0.00**	**-0.71**	**-1.04**	**-0.38**	0.68	0.06	0.16	-0.25

Statistically significant associations are in bold

As shown on Table 4, the logistic regression analysis revealed that clean water (OR = 0.05, 95%CI: 0.01-0.47) and drug availability (OR:0.23, 95%CI: 0.02-2.43) were significant risk factors after the tsunami in Aceh, and contact with other typhoid patients (OR = 20.30, 95%CI: 1.93-213.02) and education (OR = 0.08, 95%CI: 0.01-0.98) were significant after the earthquake in Yogyakarta. Under normal conditions in Bandung, we found that hand washing (OR = 0.07, 95%CI: 0.01-0.50) and education (OR = 0.08, 95%CI: 0.01-0.64) were significant risk factors.

**Table 4:** Risk factors by logistic regression analysis with dependent variable complication and non complication

	Aceh			Yogya			Bandung		
	
Risk factors		95% C.I.			95% C.I.			95% C.I.	
						
	OR	Lower	Upper	OR	Lower	Upper	OR	Lower	Upper
Age	1.01	0.91	1.01	1.02	0.94	1.11	1.12	1.01	1.23
Gender	0.74	0.05	0.74	0.37	0.03	4.41	1.13	0.22	5.86
Education	0.61	0.01	0.61	*0.08	**0.01**	**0.98**	*0.08	**0.01**	**0.64**
Recheck	0.01	0.00	0.1	1.30	0.01	121.36	1.10	0.04	27.16
Go to recheck	6.15	0.15	6.15	0.37	0.02	6.156	0.81	0.12	5.26
Drug availability	*0.23	**0.02**	**2.43**	1.22	0.06	23.21	0.35	0.07	1.64
Clean water	**0.05	**0.01**	**0.47**	0.42	0.02	6.86	0.78	0.09	6.32
Water checking	1.19	0.03	1.19	0.57	0.04	8.70	0.89	0.14	5.69
Hand washing	2.61	0.03	2.61	3.01	0.05	190.91	*0.07	**0.01**	**0.50**
Contact with other typhoid	3.15	0.12	3.15	**20.30	**1.93**	**213.02**	0.73	0.16	3.34

Significant level at P < 0.05* and P < 0.01** (Statistically significant associations are in bold)

## Discussion

The main finding of this study was that there were different characteristics and risk factors associated with typhoid complications after the tsunami in Aceh, after the earthquake in Yogyakarta, and under normal conditions in Bandung (Tables 1, Table 2). The risk factors after the tsunami were lack of clean water and availability of drugs. These factors might have increased the risk of typhoid complications at the time when the level of destruction caused by the tsunami was large [2,25,26]. The tsunami destroyed the usual sources of clean water in Aceh, e.g. wells, municipal water and other clean water sources. Therefore the proposal of developing a portable clean water device for tsunami disaster event will be useful. Transmission of *S. typhi *was introduced into the camps of IDPs as a result of their inability to carry out proper food preparation, which was associated with the need for clean water, or bacterial contamination of drinking water [1]. Consistent with this disaster situation caused by the tsunami, the independent risk factors for IDPs included problems with drug distribution and availability. This was because destruction of all the main roads, bridges, sea ports, and airports rendered surface transportation impossible, and reconstructing such facilities took considerable time. At the time, there was only one way to distribute drugs and other logistics to the IDPs camp, namely by helicopter. The lack of typhoid drugs was not caused by the type of antibiotics involved, because there was government or non-government support for the first-line drugs of choice for typhoid fever (chloramphenicol, ampicillin, ciprofloxacin and third-generation cephalosporins), but rather by the cumbersome distribution of the drugs.

**Figure 3:**
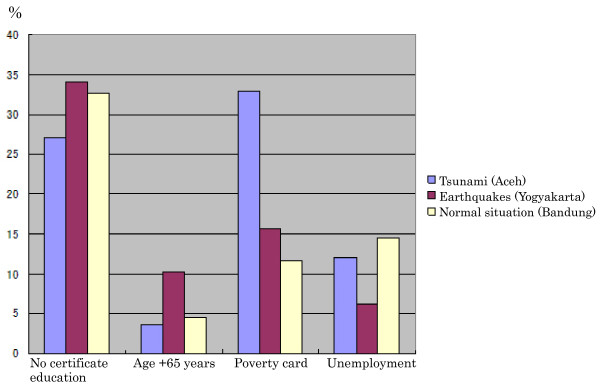
**Characteristic socioeconomic in three districts**. Source: Indonesian Health Profile 2006, Ministry of Health Indonesia.

After the earthquake in Yogyakarta, the significant risk factors included contact with other typhoid patients and education. Patients were unaware that *S. typhi *had infected their relatives or friends and this would only have emerged once the patient came to the medical facilities and was diagnosed with typhoid fever. Refer to the Indonesia Health Profile 2006 that the number of people who lacked any certificate education (not completed junior high school) in Yogyakarta was higher than in Aceh and Bandung (Figure 3). This may reflect the fact that educational level is a significant risk factor during an earthquake, as in Yogyakarta (Table 4). Therefore the rapid health promotion direct to the IDPs is necessary to reduce the risk of typhoid complication.

Under normal conditions in Bandung, we found that hand washing and education were significant risk factors. Hand washing may reflect different habits and behavior in society. The majority of those in west Java (Bandung) prefer to eat raw vegetables, [27] that sometimes are not properly washed, and followed by poor hand washing [28]. With this in mind, the government recently has promoted hand washing with soap as a national campaign in Indonesia to help prevent the infectious diseases such as typhoid complication [29]. Over 30% of persons in Bandung have no educational certificate, according to the Indonesian Health Profile (Figure 3), which also emerged consistently as a significant independent risk factor for typhoid complications.

To reach conclusions concerning the distinct risk factors in each district, we compared characteristic cases of typhoid complications in Aceh, Yogyakarta and Bandung, based on the geographical and socio-demographic differences in a typical disaster (Figure 1). One of the reasons to determine the risk factors for typhoid complications are that the first-line drugs commonly used in Indonesia have become ineffective, thus hampering treatment of the condition [17,30] and causing many complications [21-23,27] especially in disaster situation. The tsunami in Aceh was characterized by the high level of destruction, swift onrushing seawater that affected humans and the environment, the high probability of contamination of clean water, and the long time required for reconstruction/rehabilitation. The lack of clean water and available drugs were found to be related significantly to the other tsunami background factors. In comparison, during the earthquake in Yogyakarta, there was no mass of water that was likely to cause contamination to wells, municipal water and other sources of drinking water. There was no mud after the earthquake and the level of destruction was also relatively low. However, although both calamities were mainly caused by earthquakes, geographically speaking, Aceh is located on the shoreline and Bantul, Yogyakarta is located in the middle of Java Island (Figure 1). Nevertheless, the population density in Yogyakarta was the highest (255.21 per km^2^) in this study (Table 5), which means that contact with others, like relatives or friends with typhoid fever seemed more frequent in Yogyakarta than in Aceh and Bandung [12]. Although Bandung is located in the centre of West Java, it is at an elevation of 765 m above sea level, which is higher than Yogyakarta and Aceh. Surrounded by mountains, Bandung has the potential for flooding in the rainy season [14], which increases the likelihood of typhoid transmission. The educational level may also influence personal habits and efforts to avoid typhoid fever with complications, such as a lack of awareness of and/or performing hand washing properly. These differences allowed us to determine the prevalence of risk factors between tsunami, earthquake and normal situation.

**Table 5:** Demographic profile in three districts

Variables	Tsunami (Aceh)	Earthquakes (Yogyakarta)	Normal Situation (Bandung)
Population Province*	4.031.589	3.343.651	38.965.440
Pop. City/county*	220.737	813.087	2.510.982
City/county area km^2 ^*	51.937	3.186	34.597
Pop density/km^2^	4,25	255,21	72,58

*Source: BPS 2005 (Indonesian Statistical Bureau)

Patients were asked to describe their symptoms during hospitalization, as well those reported in the hospital. We found 42 patients with typhoid fever after the tsunami in Aceh, 5 of whom had complications (11.9%). Two cases each of intestinal bleeding and typhoid perforation and one of toxic typhoid were reported as complications in the post-tsunami situation, which might be caused by the lack of drug availability and clean water distribution. The number of complications in Yogyakarta was 8 of 39 cases (20.5%). We found four cases of intestinal bleeding, one of typhoid perforation and three of typhoid encephalopathy. However, there was not death case report in both of disaster situation. In Bandung, typhoid complications were found in 13 of 70 cases (18.6%), with two of intestinal bleeding, three of typhoid perforation, four of typhoid encephalopathy, and four of toxic typhoid. The death cases were due to they came to the hospital on the late stage of disease. We excluded three fatalities caused by typhoid encephalopathy (Tables 2).

As shown in Table 3, education was a significant factor in bivariate correlation analysis in the three districts. However, there is no statistical different (P = 0.42) for typhoid complication occurrence among three districts. Gasem et al in 2001 in Semarang [18] and Velema et al in 1997 in Ujung Pandang [15] reported that educational level was a significant risk factor for typhoid fever in Indonesia. A proposal to improve the quality of public education campaigns and stressing the importance of sanitation and hygiene whether in disaster or normal circumstances still appears to represent a realistic approach to infection prevention based on socio-cultural background limitation in our study.

## Conclusion

Determination of the different risk factors of typhoid fever with complications after the tsunami in Aceh (availability of clean water and drugs) and after the earthquake in Yogyakarta (education level and avoiding contact with other typhoid patients) emphasizes the need for rapid public health intervention in complex disasters compared with normal circumstances in Indonesia.

## Competing interests

The authors declare that they have no competing interests.

## Authors' contributions

AQ and HS assisted the statistical analyses and interpreting the data. TO has given a suggestion how to choose the methodology analysis and involved in revising the manuscript critically before submission. Author and co-authors have read and approved the manuscript.
